# mGem: Antibiotic tolerance across the growth-rate continuum in chronic human infections

**DOI:** 10.1128/mbio.01926-26

**Published:** 2026-10-23

**Authors:** Bethan Roberts, Sam P. Brown, Stephen P. Diggle, Marvin Whiteley

**Affiliations:** 1 Center for Microbial Dynamics and Infection, School of Biological Sciences, Georgia Institute of Technology 1372, Atlanta, Georgia, USA; Albert Einstein College of Medicine, Bronx, New York, USA

**Keywords:** antibiotics, tolerance, persisters, growth rate, resistance

## Abstract

Antibiotic failure is frequently attributed to genetic resistance, yet many chronic infections persist despite clinical isolates remaining susceptible to antibiotics. Antibiotic tolerance offers an alternative explanation, but is commonly interpreted through persisters, dormancy, and growth arrest. We argue this theory is incomplete. *In vitro* studies consistently demonstrate that growth rate strongly influences antibiotic susceptibility, while bacteria in chronic human infections occupy a broad continuum of growth states. However, experimental models of tolerance largely examine rapidly growing or growth-arrested bacteria and rarely encompass the substantially slower growth rates found in chronic infection. We propose that tolerance can emerge from stochastic growth-rate heterogeneity within clonal populations without requiring transition into dormancy. Thus, understanding tolerance requires defining how antibiotic survival changes across the growth-rate continuum. Further studies directly examining antibiotic tolerance at slow growth rates representative of chronic infection are needed to understand tolerance during human infection.

## PERSPECTIVE

Antibiotic failure is commonly attributed to genetic resistance, yet many infections relapse despite “susceptible” results during minimum inhibitory concentration (MIC) testing. This discrepancy is particularly apparent in chronic infections, such as lung infections in people with cystic fibrosis (CF), non-CF bronchiectasis, osteomyelitis, chronic wounds, and device-associated infections, where bacteria can persist despite prolonged antibiotic exposure without acquiring well-known resistance mutations (1, 2). These observations suggest that current models of antibiotic susceptibility fail to fully capture the underlying mechanisms that allow bacteria to survive during chronic human infection.

**Fig 1:**
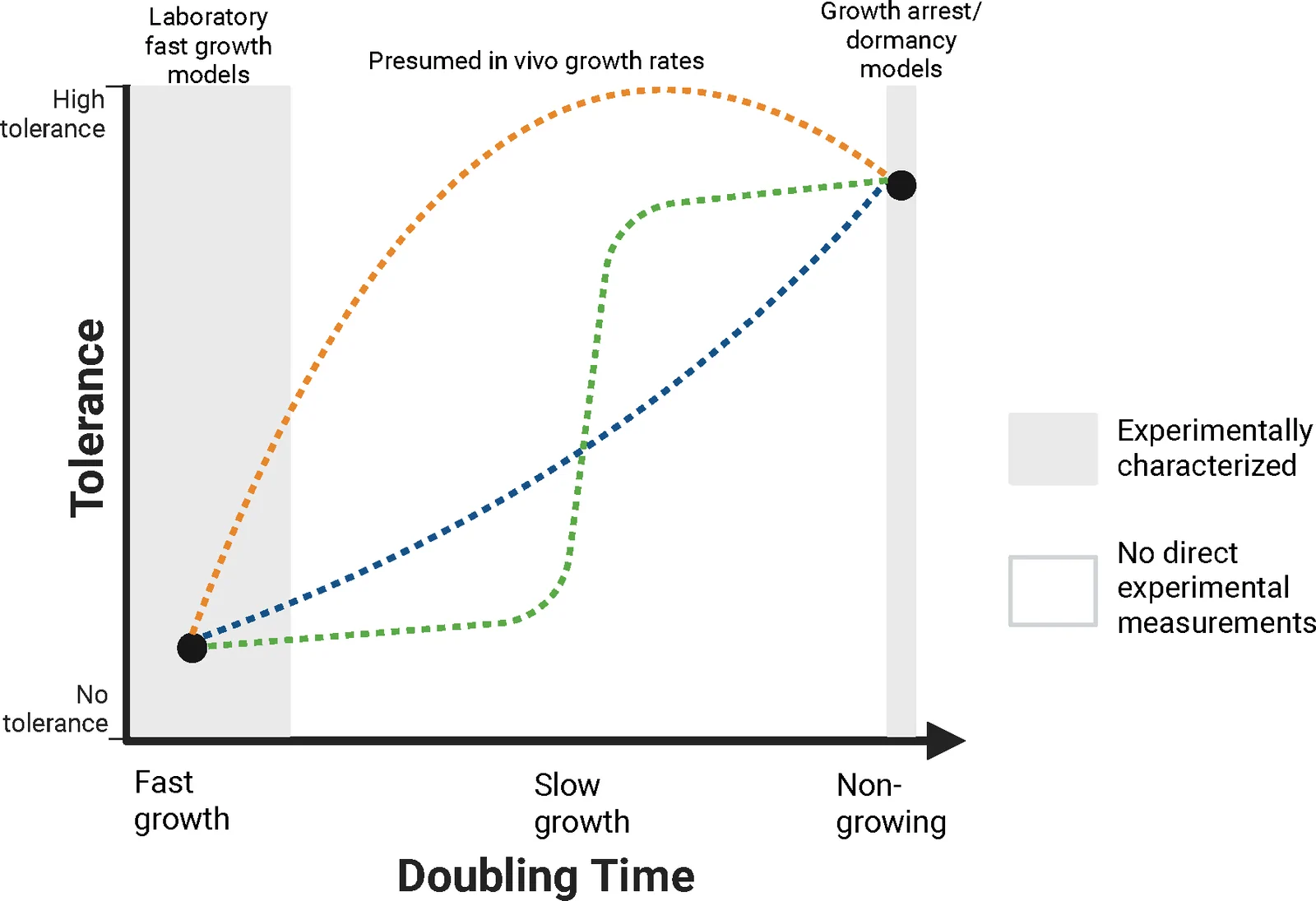
The relationship between growth rate and antibiotic tolerance during chronic infection remains undefined. The shaded regions represent current experimental evidence linking growth rate and tolerance from laboratory fast growth models (doubling times in the range of minutes to hours) and non-growing dormancy models. There is no direct experimental data for tolerance at the slow growth rates (doubling times in the range of days) theorized to exist in chronic human infection. Dashed lines illustrate possible relationships between growth rate and antibiotic tolerance, with the blue and orange lines representing different continuous relationships (monotonic or non-monotonic) and the green line representing a threshold relationship. These curves are not intended to represent specific mechanistic models, but to illustrate the range of possible relationships that remain experimentally untested.

Tolerance, defined as the ability of microorganisms to survive transient exposure to high concentrations of antibiotics *in vivo* without a change in MIC *in vitro* (3), is increasingly recognized as a key survival contributor in chronic infection (4). Unlike genetic resistance, which reflects heritable increases in MIC, tolerance arises from reversible phenotypic shifts within genetically identical populations (5). Consistent with contemporary definitions, throughout this review, we treat tolerance as the broader concept that includes persistence (1, 6). Although slow growth has long been recognized as an important factor in antibiotic survival, much of the field has interpreted tolerance through the framework of a discrete non-growing phenotypic state (persisters, dormancy, and growth arrest), leading to the prevailing hypothesis that survival primarily occurs in non-growing subpopulations (7). However, most evidence supporting this theory derives from *in vitro* models, which poorly represent bacterial physiology during infection (8, 9). Additionally, emerging evidence has suggested that a distribution of growth rates exists within an infection, rather than a binary mixture of growing and non-growing cells (10, 11). Yet, we largely do not study bacteria growing at the rates encountered during chronic infection. Experimental studies of antibiotic tolerance have focused predominantly on rapidly growing laboratory cultures or growth-arrested populations, while the substantially slower, yet actively dividing, growth rates occurring during chronic infection remain largely uncharacterized. Direct measurements linking these slow growth rates to antibiotic tolerance *in vivo* are lacking (Fig. 1).

We argue that the fundamental determinant of antibiotic tolerance is not the absence of growth but rather slow, yet still exponential, growth. Rather than referring to a specific doubling time, we use “slow growth” to describe the continuum of actively dividing physiological states between laboratory exponential growth and complete growth arrest. Our central premise is, therefore, not that slow growth has been overlooked, but that the slow growth rates relevant to chronic infection have not been adequately tested as determinants of antibiotic tolerance. The distribution of growth rates within an infection does not reflect coexisting strains with different intrinsic growth rates, but rather stochastic variation in growth rate within a population of a single strain. As a result, a single genotype can produce a range of subpopulation tolerances depending on specific subpopulation growth rates. Thus, net population-scale tolerance is expected to emerge as a natural byproduct of growth rate heterogeneity as opposed to being a discrete physiological state entered only by a subset of specialized cells. Slow-growth-induced tolerance could scale smoothly with doubling time or could be linked to a specific growth rate threshold (or thresholds) that trigger increasing tolerance (Fig. 1). Figure 1 flags the core gap in our data and understanding regarding the response of slow-growing cells to antibiotics. Identifying the full continuum of growth rates in chronic infection and their growth-specific influence on survival is a crucial challenge for the field. We contest the common assumption that survival is primarily associated with growth arrest and instead advocate that changes in the growth dynamics of exponential cells can be sufficient to initiate survival during antibiotic exposure.

**Fig 2:**
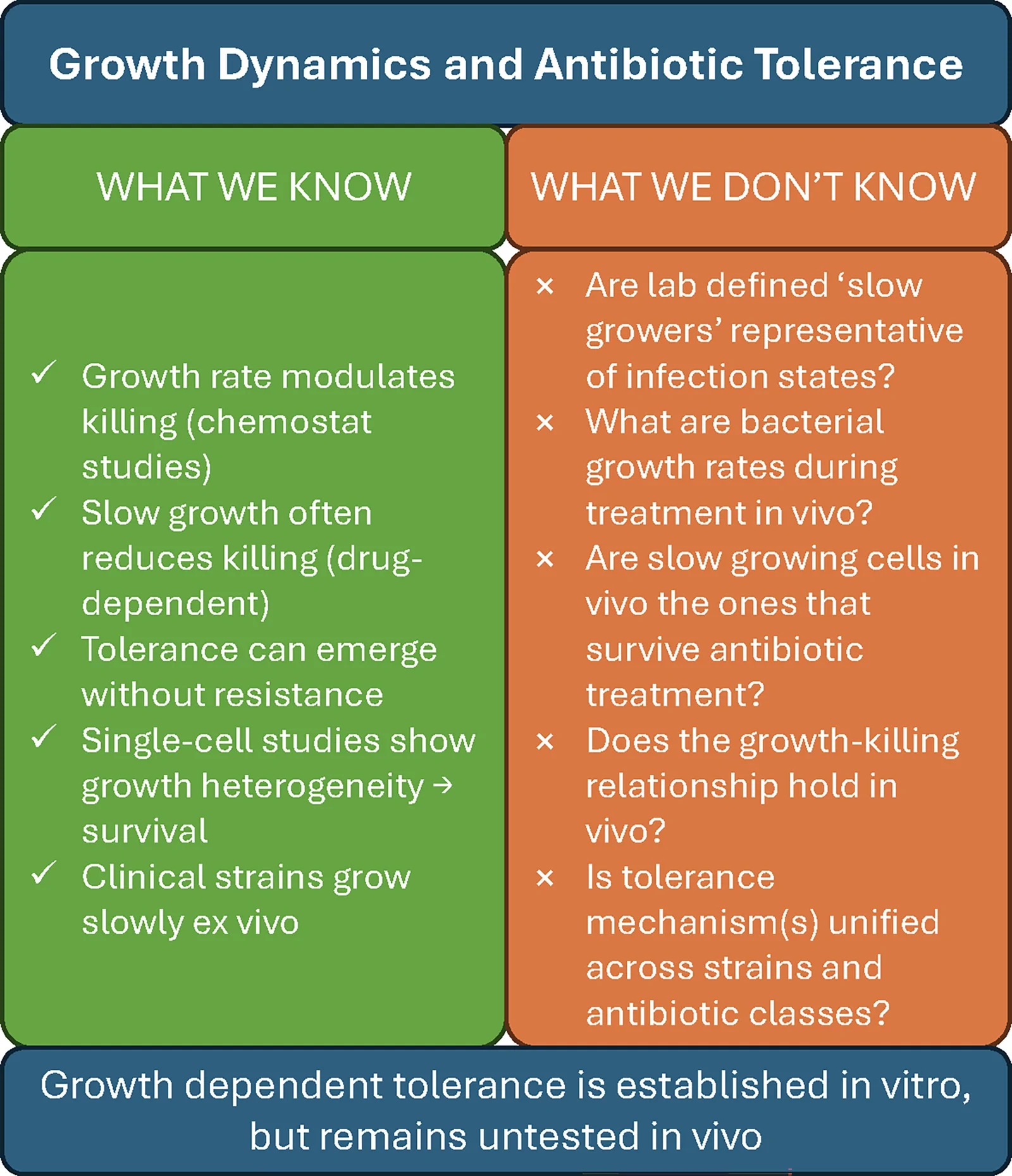
What we know and what remains unsolved about growth-mediated antibiotic tolerance.

In this mGem, we reconsider antibiotic tolerance through the lens of chronic human infection. We review evidence linking bacterial growth dynamics to antibiotic killing, examine what is currently known about bacterial growth during infection, and highlight the major unresolved question of whether specific *in vivo* growth states directly determine survival during antibiotic treatment (Fig. 2). While growth-mediated tolerance is well supported *in vitro*, its relevance and the effect of growth rate in human infection remain largely inferred rather than directly demonstrated. A central priority is, therefore, to experimentally define antibiotic survival across the growth rates experienced by bacteria during chronic human infection.

## GROWTH RATE AS A DETERMINANT OF ANTIBIOTIC TOLERANCE

Numerous mechanisms have been proposed to explain how bacteria survive antibiotic exposure without the presence of classical resistance mutations. Many proposed tolerance mechanisms involve physiological adaptations targeting cellular growth or metabolic activity, including persister formation, biofilm growth, and stress-associated metabolic remodeling (12, 13). However, because many bactericidal antibiotics depend on active processes, such as cell wall synthesis, DNA replication, or translation, slowing these processes can substantially reduce antibiotic efficacy without altering MIC, explaining why dormancy became the prevailing hypothesis for tolerance (14). Experimental evidence linking bacterial growth rate to antibiotic susceptibility first emerged from chemostat studies, where nutrient availability and division rate can be controlled independently of other environmental variables. In both *Escherichia coli* and *Streptococcus pneumoniae*, progressively reduced growth rates consistently decreased susceptibility to β-lactam antibiotics (15–18). Because β-lactams target active cell wall synthesis, their bactericidal activity declines as growth rate slows, and peptidoglycan turnover decreases (19). These early studies established the fundamental principle that antibiotic efficacy is not fixed but instead is subject to the physiological state of cells.

Subsequent work expanded this principle beyond cell wall-targeting antibiotics. Systematic analyses across multiple bacterial species and drug classes demonstrated that bacterial growth rate often modulates antibiotic killing, although the magnitude and direction of the effect depend on both bacterial physiology and antibiotic mechanism of action (20). This study further demonstrated that while suboptimal growth rate frequently reduced killing, several drug species combinations exhibited comparable or even enhanced killing under suboptimal growth conditions, indicating that growth-mediated tolerance is not universally conserved across all antimicrobial classes.

More recent single-cell and quantitative approaches refined this model. In *Salmonella enterica*, disruption of known persister-associated pathways, including (p)ppGpp signaling, toxin–antitoxin systems, ATP depletion, and acidic stress responses (21–24), failed to stop tolerance to ciprofloxacin and ceftriaxone (25). Instead, reduced growth itself emerged as the primary determinant of survival in *S. enterica*, consistent with the inverse relationship between growth and antibiotic survival reported in *E. coli* and *Staphylococcus aureus* (26–28). Time-lapse microscopy in *E. coli* further demonstrated that extended lag times before cell division strongly correlate with survival during antibiotic exposure (29), while quantitative modeling of ribosome-binding antibiotics showed that growth rate and drug-binding kinetics together can predict susceptibility across nutrient conditions (30). Collectively, these *in vitro* studies demonstrate that manipulation of growth dynamics alone can substantially alter antibiotic susceptibility without any genotypic changes.

## EVIDENCE FOR SLOW BACTERIAL GROWTH IN HUMAN INFECTIONS

Following *in vitro* evidence that reduced growth promotes antibiotic tolerance, several studies have examined bacterial growth directly in clinical samples from chronic lung infections. In patients with CF, where *Pseudomonas aeruginosa* can persist for decades, fluorescence *in situ* hybridization (FISH) targeting ribosomal RNA (rRNA) has been used to estimate bacterial growth directly from sputum samples (31, 32). *In situ* measurements revealed that most cells doubled on timescales of 100–200 min, considerably slower than under standard laboratory conditions, and identified considerable heterogeneity in ribosome content consistent with the presence of a broad range of growth rates (31). However, rRNA abundance becomes increasingly decoupled from cell division at very low growth rates, limiting the ability of FISH-based approaches to distinguish slow-growing from non-growing populations (33, 34). Stable isotope labeling methods have been applied to sputum samples to provide an independent measure of bacterial growth. Using heavy water incorporation into DNA, *S. aureus* in CF sputum was estimated to divide on timescales of days under *ex vivo* conditions (35, 36). Notably, these *ex vivo* growth rates extend well beyond those typically examined in laboratory models of antibiotic tolerance. Together, these studies indicate that bacterial growth during chronic lung infection is markedly constrained compared with laboratory culture conditions, likely generating a heterogeneous population spanning a continuum of growth rates.

Importantly, none of these studies directly measured antibiotic killing within the lung. However, both *P. aeruginosa* and *S. aureus* isolated from CF patients frequently exhibit pronounced antibiotic tolerance in clinical settings, surviving prolonged antibiotic exposures that can exceed *in vitro* MIC values by thousands-fold despite limited classical resistance (37, 38). In parallel, many clinical isolates exhibit intrinsically slow-growing phenotypes that persist under laboratory conditions, which revert alongside increased antibiotic susceptibility as growth rate increases (39–41). These observations support a central role for reduced growth in antibiotic tolerance, although direct experimental evidence between growth rate and survival during treatment *in vivo* remains to be demonstrated. However, they also challenge the assumption that chronic infections are dominated exclusively by dormant bacteria, especially as sustained infection despite frequent expectoration implies ongoing bacterial replication, even if substantially slowed.

## DOES SLOW GROWTH DRIVE ANTIBIOTIC TOLERANCE *IN VIVO*?

Although slow bacterial growth and antibiotic recalcitrance coexist during chronic infection, directly linking these phenomena remains challenging. Experimental infection models provide some evidence that tolerance can emerge within host environments. In *Galleria mellonella* infection models, *P. aeruginosa* populations surviving antibiotic treatment exhibited both increased minimum duration to kill and reduced growth rates without changes in MIC (42). Similarly, murine chronic lung infection models demonstrated that slow-growing isolates survive antibiotic therapy more effectively *in vivo* (43). Clinical observations further support the relevance of growth-linked tolerance during infection. Slow-growing phenotypic variants, such as *S. aureus* small colony variants, are frequently recovered from chronic infections, including osteomyelitis and device-associated infections, and associated with reduced susceptibility to antibiotic killing despite lacking classical resistance mutations (44). Together, these findings indicate that phenotypic tolerance can arise and persist during infection and contribute to treatment failure.

## CONCLUSION

Despite substantial experimental progress, the central hypothesis that slow-growing cells drive antibiotic tolerance during human chronic infection remains largely untested (Fig. 2). Growth rate and antibiotic survival are rarely measured simultaneously within infected tissues, and most tolerance phenotypes are concluded from isolates removed from their host environment. Consequently, it remains unclear whether bacteria identified as slow growing *in vitro* accurately reflect the physiological states that survive antibiotic treatment *in vivo*. Current models, therefore, provide little insight into how antibiotic killing changes across the range of growth rates observed during chronic infection despite evidence that bacteria can divide on timescales ranging from hours to days. Answering this question requires moving beyond binary classifications of growing and non-growing cells and instead understanding how antibiotic survival changes across the distribution of growth rates during infection. Defining the growth-killing relationship *in vivo* will reveal whether treatment failure is driven primarily by a discrete persister compartment or by the wider distribution of slow growing cells and therefore whether therapy must target a specialized state or a physiological continuum.
